# Muscle diffusion MRI reveals autophagic buildup in a mouse model for Pompe disease

**DOI:** 10.1038/s41598-023-49971-9

**Published:** 2023-12-20

**Authors:** Marlena Rohm, Gabriele Russo, Xavier Helluy, Martijn Froeling, Vincent Umathum, Nicolina Südkamp, Denise Manahan-Vaughan, Robert Rehmann, Johannes Forsting, Frank Jacobsen, Andreas Roos, Yoon Shin, Anne Schänzer, Matthias Vorgerd, Lara Schlaffke

**Affiliations:** 1 Department of Neurology, Berufsgenossenschaftliches-University Hospital Bergmannsheil gGmbH, Ruhr-University Bochum, Bürkle-de-la-Camp-Platz 1, 44789 Bochum, Germany; 2 Heimer Institute for Muscle Research, BG-University Hospital Bergmannsheil gGmbH, 44789 Bochum, Germany; 3 Department of Neurophysiology, Medical Faculty, Ruhr-University Bochum, 44801 Bochum, Germany; 4 Department of Biopsychology, Institute of Cognitive Neuroscience, Faculty of Psychology, Ruhr-University Bochum, 44801 Bochum, Germany; 5 Department of Radiology, University Medical Centre Utrecht, 3584 CX Utrecht, The Netherlands; 6 Institute of Neuropathology, Justus Liebig University, 35390 Giessen, Germany; 7 Institute of Pathology and Molecular Pathology, Bundeswehrkrankenhaus Ulm, 89081 Ulm, Germany; 8 Department of Neuropediatrics, University Hospital Essen, Duisburg-Essen University, 47057 Essen, Germany; 9 Molecular Genetic and Metabolism Laboratory, 80333 Munic, Germany; 10 University Children’s Hospital, 80333 Munich, Germany

**Keywords:** Preclinical research, Neuroscience

## Abstract

Quantitative muscle MRI is increasingly important in the non-invasive evaluation of neuromuscular disorders and their progression. Underlying histopathotological alterations, leading to changes in qMRI parameters are incompletely unraveled. Early microstructural differences of unknown origin reflected by Diffusion MRI in non-fat infiltrated muscles were detected in Pompe patients. This study employed a longitudinal approach with a Pompe disease mouse model to investigate the histopathological basis of these changes. Monthly scans of Pompe (Gaa^6neo/6neo^) and wildtype mice (age 1–8 months) were conducted using diffusion MRI, T2-mapping, and Dixon-based water-fat imaging on a 7 T scanner. Immunofluorescence studies on quadriceps muscles were analyzed for lysosomal accumulations and autophagic buildup and correlated with MRI outcome measures. Fat fraction and water-T2 did not differ between groups and remained stable over time. In Pompe mice, fractional anisotropy increased, while mean diffusivity (MD) and radial diffusivity (RD) decreased in all observed muscles. Autophagic marker and muscle fibre diameter revealed significant negative correlations with reduced RD and MD, while lysosomal marker did not show any change or correlation. Using qMRI, we showed diffusion changes in muscles of presymptomatic Pompe mice without fat-infiltrated muscles and correlated them to autophagic markers and fibre diameter, indicating diffusion MRI reveals autophagic buildup.

## Introduction

Over the past years, non-invasive quantitative MRI (qMRI) sequences have provided the possibility to sensitively monitor disease progression in neuromuscular disorders. For example, Dixon-based imaging can monitor fatty degeneration over time^1–3^. An increase in water-T2 is associated with inflammation and has therefore been proposed as an objective measure of disease activity in diseases such as Duchenne muscular dystrophy^4^. More recent use of other qMRI methods, such as diffusion tensor imaging (DTI), demonstrate that even healthy-appearing muscles in terms of a lack in fat infiltration or atrophy in late-onset Pompe disease (LOPD) show significant differences in diffusion parameters, indicating early microstructural alterations^5^.

This finding is of great importance due to the fact that fatty degeneration is irreversible, while microstructural alterations might still be reversible^6,7^. Therefore, disease-modifying therapies should aim at preserving healthy muscle tissue. Thus, the understanding of microstructural muscle alterations from lysosomal dysfunction and autophagic buildup, and their respective correlations to qMRI, such as DTI-based mean diffusivity (MD) and fractional anisotropy (FA), values are crucial, both to define an optimal timepoint for possible therapy and to develop novel therapeutic targets. To allow a comprehensive and longitudinal analysis of underlying histopathological mechanisms of upper and lower leg muscles, the translation to a mouse model is needed. One example is the Gaa^6neo/6neo^ mouse, a model for the morbus Pompe disease that allows the correlation of histopathology to qMRI parameters in a controlled setup. Pompe disease is a hereditary glycogen storage disease where a defect in the lysosomal enzyme alpha-glucosidase (GAA) leads to progressive lysosomal glycogen overload predominantly in striated muscles and motor neurons^8,9^. Histologically, skeletal muscles of Popmpe patients are characterized by vacuolar fibres resulting from the accumulation of enlarged glycogen-filled lysosomes^10^. In addition, massive autophagic buildup occurs due to altered autophagy and dysfunctional lysosomes^11^.

Regularly administered intravenous enzyme replacement therapy (ERT) using recombinant human GAA, can slow down muscle degeneration and disease progression. The ideal starting point for ERT in LOPD—either before clinical manifestation or when patients get symptomatic—remains unclear^12^.

We hypothesize that diffusion changes in presymptomatic skeletal muscles in Pompe disease is caused by either lysosomal glycogen, or autophagic buildup. To address this assumption, this study used a qMRI scanning protocol, compromising DTI, T2 mapping and Dixon-based MRI, for mouse muscle in the hind limb, equivalent to MR protocols used for patients^13^. qMRI data from the pre-symptomatic mice at different ages were correlated with results from muscle histology to explain dynamic changes in qMRI parameters.

## Methods

### Standard protocol approvals

All animal experiments were performed following the German Law on the protection of animals (TierSchG §§ 7–9) and were approved by a local ethics committee LANUV, North-Rhine-Westphalia, Germany, 81–02.04.2021.A169). The involved authors followed the ARRIVE guidelines.

### Study design

**Figure 1:**
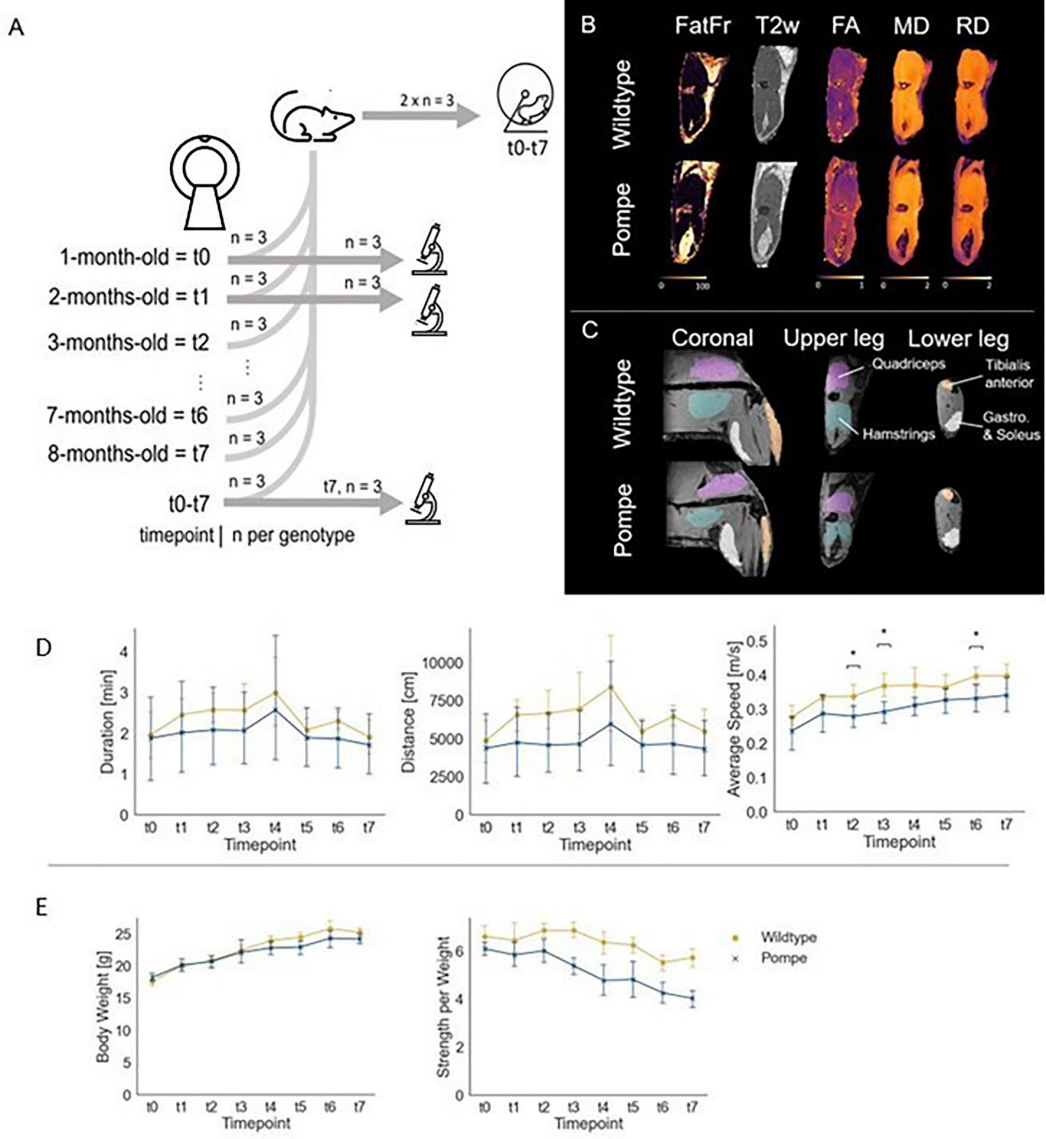
(**A**) Schematic representation of the study design. Three mice per genotype were scanned at each timepoint, with histological studies carried out with mice scanned at t0, t1 and t7. In addition, one group of three mice per genotype was scanned continuously from t0-t7. Two groups of each three mice per genotype were housed monthly in an IntelliCage system equipped with running wheels. (**B**) Exemplary images of processed data for mice at the age of 8 months (t7). From left to right: fat fraction (FatFr), water-T2 (T2w), fractional anisotropy (FA), mean diffusivity (MD), radial diffusivity (RD). (**C**) Manual segmentation with underlying outphase image. Coronal cut to visualize the placement of the mice with upper and lower leg segmentations. Labels are as indicated. (**D**) Phenotyping with IntelliCage. Mean duration, mean distance and average speed of runs in a session showed no significant differences over group and time. However average speed in Pompe mice was significantly lower at timepoint t2,t3 and t6 compared to wildtype. (**E**) The mean body weight of Pompe and wildtype mice was measured throughout the study. With n = 6–15 per genotype per timepoint, the body weight showed no significant difference. Grip strength measurement was normalized to body weight. With n = 6–12 per genotype per timepoint, grip strength revealed no significant difference.

This study was designed to (i) monitor changes in muscles using qMRI and (ii) correlate qMRI findings with muscle histology in the hind limb of Pompe mice compared to wildtype mice between one and eight months of age (Fig. 1A). A total of 27 Pompe and 27 wildtype mice were examined with qMRI, scanning the left hind limb. The protocol, adapted from patient studies, included Dixon-based imaging for fat quantification, water-T2 mapping imaging for oedematous and inflammatory representation, and DTI for detecting microstructural changes^13,14^. A first cohort of three Pompe and three wildtype mice was scanned continuously every month from the age of one to eight months. The second cohort consisted of three mice per genotype and timepoint. Taken together, both cohorts yielded a total of six mice per genotype and timepoint for MRI analysis. Since this study aimed to unravel the underlying origins of early diffusion changes, three timepoints were further analyzed for histopathological analysis and correlated with qMRI: t0—no significant differences in diffusion parameters between Pompe and wildtype mice; t1—first significant differences in FA and RD between Pompe and wildtype mice; t7—Pompe mice display a phenotype with differences in muscle function according to relevant literature^15^. To assess the phenotype of both Pompe and wildtype mice, their activity in running wheels, grip strength and weight were recorded monthly (n = 6 per genotype).

### Animals

The animal model Gaa^6neo/6neo^ used in this study has a target disruption in exon 6 in the *GAA*-gene resulting in the lack of enzyme activity of GAA^15^. The assay of acid alpha glucosidase activity was performed on soleus muscle samples for three timepoints (0, 1 and 7) for each wildtype and pompe mice by a fluorometric method using 4-Methylumbelliferyl-alpha-glucoside (4MUG) as the substrate. About 1 mg muscle biopsy was homogenized in 150 µL 0.2 M Acetate buffer, pH 4.0. and the reaction mixture contains 2.5 mM 4MUFG in 0.2 M Acetate buffer. Female Gaa^6neo/6neo^ (Jackson Laboratory, B6; 129-Gaatm1Rabn/J) and wildtype mice at the age of four weeks to eight months were housed in a controlled environment (light/dark cycle 12/12h; 22 °C; food/water ad libitum). Only female mice were used in this experiment to avoid any territorial and aggressive behaviour when performing the phenotyping in the joint intellicage housing.

### Phenotyping

For one week each month, Pompe and wildtype mice (t0–t7; n = 3 per genotype, two iterations) were housed in an IntelliCage (TSE Systems, Germany) equipped with three running wheels. An RFID chip was implanted into the neck of the mice at the age of four weeks while being anaesthetized with isoflurane. While using running wheels, duration, speed and distance covered were recorded. Data were analyzed by calculating the mean duration, speed and distance of all sessions for each mouse, while one session was defined as one entry and exit via the gates towards the running wheels. The grip strength of the forelimb was measured with Grip Strength Meter V2.5.1 (TSE Systems, Germany) and the weight was noted monthly (t0-t7).

### MRI protocol

Imaging was performed using a small animal MRI system (7T horizontal bore, 70/30 USR, Bruker BioSpec, Germany). An 80 mm transmit/receive quadrature birdcage resonator was used for radio frequency transmission and a single-loop 20 mm surface coil for signal detection. ParaVision 6 was used to acquire the data. After initial anaesthesia (3% isoflurane in an air and oxygen mixture at a 4:1 ratio for 3 min), a thin layer of D-Panthenol (Bepanthen eye and nose ointment, Bayer, Germany) was spread over the eyes, ensuring hydration. Mice were placed on a heated flat surface, custom 3D-printed to accommodate the surface coil at leg level. The left leg was positioned to have an angle of around 90° between the tibia and femur (Fig. 1C). Anesthesia (1.7% isoflurane) and normothermia were maintained throughout the experiment.

The protocol was performed as previously described, translated from humans to rodents with an approximate scanning time of 1 h (Table 1)^13^. In brief, the left upper and lower leg was scanned separately with diffusion-weighted imaging (DWI) for DTI analysis and water-T2 mapping. Slices were positioned orthogonal to the muscle fibres starting around 3 mm from the knee joint. DWI was performed using a single-shot echo planar imaging (EPI) multi-shell Stejskal–Tanner sequence, whereas water-T2 mapping was derived from a multi-slice multi-echo (MSME) sequence. Subsequently, the whole leg was scanned with a 3D Multi Echo Gradient Echo acquisition (MEGE) to perform Dixon-based fat quantification.

**Table 1:** Scan parameter for water-T2, diffusion-weighted imaging (DWI) and Dixon-based sequences.

Sequence	Water-T2 mapping	DWI	Dixon-based imaging
	MSME	Single-shot EPI	MEGE
FOV (mm^3^)	18.3 × 15.6 × 5.25	18 × 10 × 5.25	20 × 15.6 × 20
Voxel size (mm^3^)	0.15 × 0.15 × 0.75	0.2 × 0.2 × 0.75	0.2 × 0.2 × 0.2
Slice Gap (mm)	0.25	–	–
Slices	7	7	50
TR (ms)	2200	2000	200
TE (ms)	4.7–84.9	18.06	1.9–19
Echo spacing (ms)	4.71	–	1.904
b-values s/mm^2^ (number of gradients)	0	0 (n = 43), 10 (n = 18), 25 (n = 18), 100 (n = 18), 200 n = 36), 400 (n = 48), 600 (n = 72) Total number of gradient orientations: 253	0
Diffusion time	–	δDuration 3 ms ∆Separation 10.04 ms	
Fat suppression	–	CHESS (Fat-Sat)	–

### MRI data processing

Data processing was adapted from previous human muscle studies using QMRITools (http://www.qmritools.com/ on Mathematica 13)^13^. In brief, for preprocessing of Dixon-based data, the IDEAL method was used, resulting in a fat fraction map and reconstructed in- (IP) and out-phase (OP) images^16^. Water-T2 was fitted with the extended phase graph fitting approach, which fits both water and fat separately using a two-compartment model^17^. Acquired DWI data were processed to generate DTI maps. In detail, data were denoised with the PCA method and corrected for motion and eddy currents. Subsequently, the tensors were calculated by taking IVIM into account and using the iWLLS algorithm^18,19^. Three eigenvectors v_1_-v_3_ and the eigenvalues λ_1_–λ_3_ were calculated from the tensor for each voxel. Based on the eigenvalues, maps for fractional anisotropy (FA), mean diffusivity (MD) and radial diffusivity (RD) were obtained (Fig. 1B).

Two wildtype datasets had to be excluded from DWI analysis, due to heavy motion artefacts that occurred during acquisition at t6.

Tibialis anterior, gastrocnemius with soleus, quadriceps and hamstring muscles were manually segmented with 3D slicer (v4.4, https://www.slicer.org/) on OP images according to anatomical templates in a mouse atlas (Fig. 1C). Segmentations were eroded by one voxel and automatically aligned to DTI and T2 space.

### Glycogen assay

Frozen quadriceps samples (0.2 mg/µl, t0/t1/t7; each n = 3 per genotype/timepoint) were homogenized in water and boiled at 95 °C for 5 min. After centrifugation at 13,000*g* for 5 min at 4 °C, the supernatant was compared against glycogen standards using the colourimetric protocol outlined in the Glycogen Assay Kit (MAK016, Sigma Aldrich, USA) and measured at 570 nm.

### PAS-staining on semithin sections and morphometric analysis

Small samples of quadriceps muscles (t0/t1/t7; n = 3 per genotype/timepoint) were fixed in 4% glutaraldehyde/0.4M PBS and processed as previously described^10,20^. In brief, 1–2 µm semithin sections were cut from resin-embedded tissue and stained with Periodic acid-Schiff (PAS).

To measure the diameter of muscle fibres, regions of interest (ROI) between 0.08 and 0.46 mm^2^ per section were selected. False positives were manually excluded. Using the NDP.view2 software (U12388-01) the diameter of each fibre within the ROI was measured.

PAS-positive deposits were analyzed with QuPath software^21^. In two ROIs per section, PAS-positive structures were annotated and false positives were removed manually (t0 n = 3, t1/t7 n = 2 per genotype/timepoint). The number of PAS-positive structures was counted automatically and converted into positive objects per mm^2^ for each section. The percentage of PAS-positive structures per ROI was expressed by positive pixel area per ROI.

### Immunofluorescence studies

Quadriceps muscles (t0/t1/t7; n = 3 per genotype/timepoint) were snap-frozen in liquid-nitrogen-cooled isopentane. Cryosections of 5 µm thickness were fixed with acetone and blocked with 10% goat serum/1% BSA. Primary antibodies for LC3, p62 and LAMP1 were incubated with anti-DYS2 overnight (see Table S1). After washing with PBS, secondary antibodies were incubated for 1 h. Three random equal-sized ROIs were selected and analyzed with thresholding in Fiji (v2.9.0) to calculate positively stained areas^22^.

### Statistical analysis

All statistics were conducted using python (v 3.7) with statsmodel (v0.13.2), statannot (v0.2.3) and scipy (v1.7.3). Figures were created with seaborn (v0.12.1) and GraphPad Prism (v9.4.1).

To compare the influence of age (t0-t7) and genotype (Pompe or wildtype) on the variables of interest, a two-way ANOVA was used for longitudinal data. If ANOVA was significant, a post hoc t-test was carried out to compare genotypes at single timepoints (*p < 0.05, **p < 0.01, ***p < 0.001, ****p < 0.0001).

Pearson correlation on diffusion parameter, the abundance of fluorescence and fibre size was carried out with mice sacrificed after MRI at t0, t1 and t7 (n = 3 per genotype and timepoint).

## Results

### Phenotyping with IntelliCage showed no significant differences over time and group until t7

The GAA activity assay revealed normal activity for the three tested wildtype samples (0.71, 0.80, 0.87 nmol/min/mg protein) and reduced GAA activity for the three tested Pompe mice (< 0.01, 0.012, 0.017 nmol/min/mg protein).

The phenotype of Pompe mice from t0 to t7 was observed in the IntelliCage, which recorded activity in running wheels. No significant differences between Pompe and wildtype mice were observed when duration, distance or average speed of runs over the timecourse were compared (Fig. 1D; two-way ANOVA with group and timepoint, all p > 0.94). However, Pompe mice showed a lower average speed in general with significance at timepoints t2, t3 and t6 (t0 p = 0.26, t1 p = 0.17, t2 p = 0.04, t3 p = 0.02, t4 p = 0.11, t5 p = 0.24, t6 p = 0.04, t7 p = 0.13).

Although Pompe mice showed a lower increase of body weight at t4 when compared to wildtype (F(1,7) = 1.32, p = 0.25), the development did not differ significantly. Pompe mice showed insignificant lower grip strength per weight than wildtype mice, however (F(1,7) = 1.47, p = 0.18). See Fig. 1E.

### Quantitative MRI revealed significant diffusion changes in Pompe mouse muscle starting from t1

**Figure 2:**
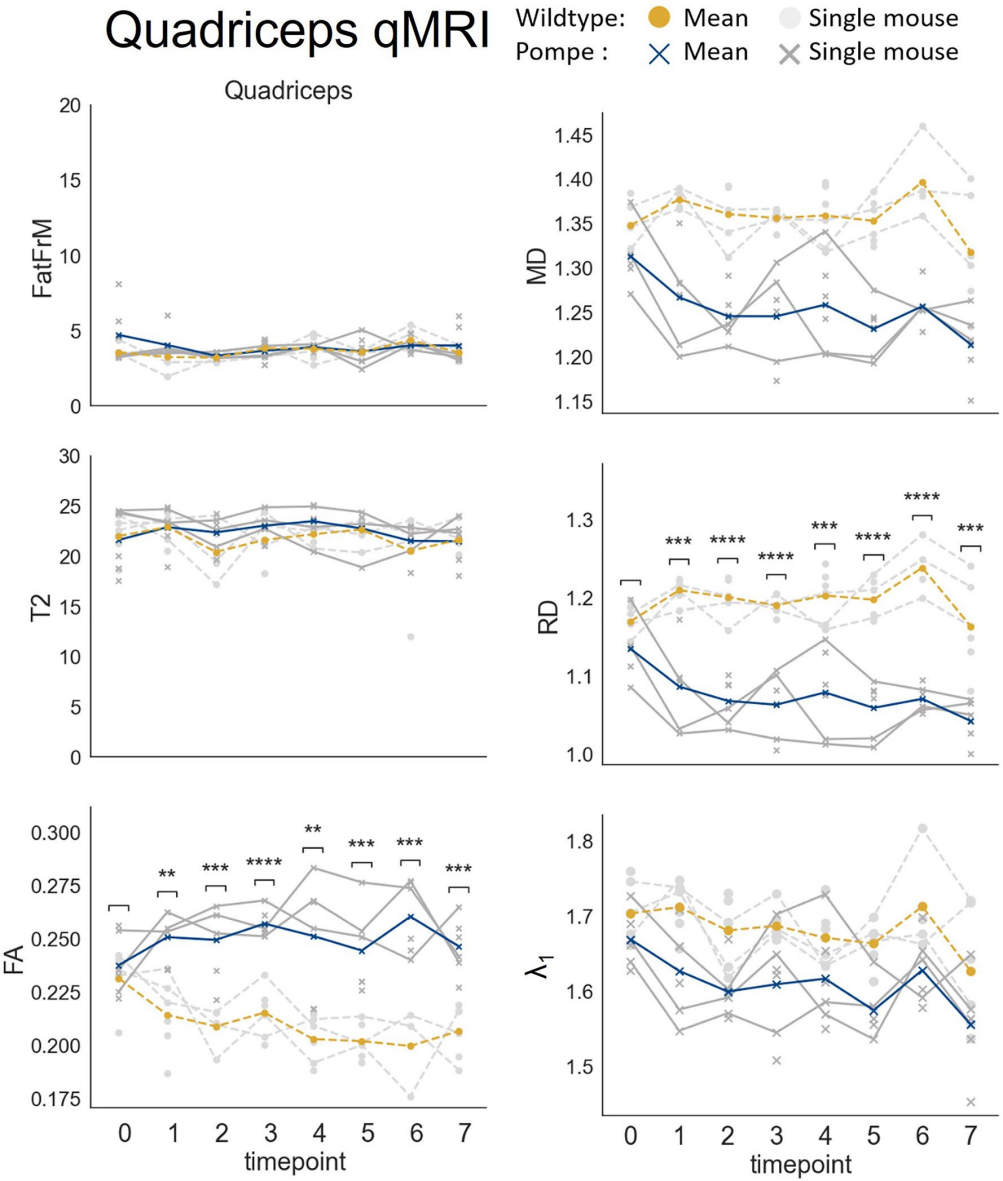
qMRI results for the quadriceps muscle (n = 4–6 per genotype and timepoint). Dots indicate wildtype mice, while crosses indicate pompe mice (grey = single timepoint, orange/blue = mean). Datapoints of mice that were continuously monitored over time using MRI are connected with a solid line for pompe mice and a dashed line for wildtype mice. Unconnected dots and crosses indicate the mice that were scanned just once and sacrificed subsequently. Fractional anisotropy (FA) and radial diffusivity (RD) showed a significant change over time, while water-T2 mapping (T2), the fat fraction (FatFr), mean diffusivity (MD) and λ1 did not. Asterisks indicate significance comparing Pompe and wildtype within timepoints.

Longitudinal qMRI measurements were carried out on the left hind limbs of the Pompe and wildtype mice from t0 to t7. As a representative overview, the results for the quadriceps are displayed in Fig. 2, with mean values per timepoint given in Table S2. For significance levels of ANOVA with respective t-tests see Table 2.

**Table 2:** Statistical analysis of qMRI parameters fat fraction (FatFr), water-T2 mapping (T2), fractional anisotropy (FA), mean diffusivity (MD), radial diffusivity (RD) and λ1.

Muscle	tp	FatFr		T2		FA			MD			RD			λ_1_	
		Anova		Anova		Anova		T-test	Anova		T-test	Anova		T-test	Anova	
		F-val	p-val	F-val	p-val	F-val	p-val	p-val	F-val	p-val	p-val	F-val	p-val	p-val	F-val	p-val
Quadriceps	t0	3.00	0.09	0.00	0.95	9.80	< 0.01	0.458	3.53	0.06	-	7.19	0.01	0.074	0.17	0.68
	t1							0.002						< 0.001		
	t2							< 0.001						< 0.001		
	t3							< 0.001						0.003		
	t4							0.003						< 0.001		
	t5							< 0.001						< 0.001		
	t6							0.001						< 0.001		
	t7							< 0.001						< 0.001		
Hamstrings	t0	0.67	0.42	0.53	0.47	14.53	< 0.01	0.131	6.58	0.01	0.296	12.13	< 0.01	0.137	0.17	0.68
	t1							0.108			< 0.001			< 0.001		
	t2							0.007			< 0.001			< 0.001		
	t3							0.003			0.005			< 0.001		
	t4							< 0.001			0.005			< 0.001		
	t5							< 0.001			< 0.001			< 0.001		
	t6							0.002			< 0.001			0.001		
	t7							< 0.001			0.002			< 0.001		
Gastro. + Soleus	t0	0.01	0.92	0.00	0.98	7.49	0.01	0.319	7.50	0.01	0.810	9.41	< 0.01	0.593	3.19	0.08
	t1							0.010			< 0.001			< 0.001		
	t2							< 0.001			< 0.001			< 0.001		
	t3							0.002			0.011			0.006		
	t4							0.004			0.002			0.002		
	t5							0.021			0.005			0.008		
	t6							< 0.001			< 0.001			< 0.001		
	t7							< 0.001			0.002			< 0.001		
Tibialis anterior	t0	0.00	0.97	0.03	0.86	13.61	< 0.01	0.041	11.48	< 0.01	0.574	16.95	< 0.01	0.273	3.43	0.07
	t1							< 0.001			< 0.001			< 0.001		
	t2							0.002			< 0.001			< 0.001		
	t3							< 0.001			0.009			< 0.001		
	t4							< 0.001			< 0.001			< 0.001		
	t5							0.014			0.026			0.022		
	t6							< 0.001			< 0.001			< 0.001		
	t7							< 0.001			< 0.001			< 0.001		

When two-way ANOVA (timepoint, group) was significant, t-tests were carried out.

The left hind limb muscles showed no significant dynamic in fat fraction and water-T2 when Pompe and wildtype mice were compared using a two-way ANOVA with factor time and group (fat fraction: all muscles in range F(1,7) = 0–3, p > 0.09; water-T2: all muscles in range F(1,7) = 0–0.53, p > 0.47). The mean fat fraction over all muscles was 3.51 ± 1.01% and 3.31 ± 0.92% for Pompe and wildtype mice respectively. The mean water-T2 over all muscles was 21.04 ± 2.36 ms and 20.44 ± 2.28 ms for Pompe and wildtype mice respectively. Both parameters did not differ significantly between groups.

Diffusion parameters FA, MD and RD were significantly different in upper and lower leg muscles when Pompe and wildtype mice were compared over 8 months (t0–t7; for quadriceps see Fig. 2, for other muscles see Fig. S1). While no significant difference occurred at t0, from t1 onwards a highly significant increase in FA (p < 0.01) and a decrease in RD (p = 0.01) in Pompe muscles were evident. MD also decreased significantly in the tibialis anterior, gastrocnemius and hamstring muscles but not in the quadriceps muscle of Pompe mice. The development of λ_1_ did not differ significantly between Pompe and wildtype mice, although it decreased in Pompe mice after t1.

### Histopathological studies at t0, t1 and t7

Given that upper leg muscles are primarily affected in the majority of the LOPD patients^5,23^ and the dissection of the quadriceps can be performed more accurately compared to the hamstrings, the quadriceps muscle was used for further histological analysis. Wildtype mice showed no quantifiable positive staining for PAS, p62, LC3 or LAMP1, therefore quantitative analysis was performed only on Pompe mice.

### Glycogen concentration was highly increased over the observation time course

Biochemical measurement of glycogen concentration showed significantly high and stable glycogen concentrations over the time course, starting at t0 in the quadriceps of Pompe mice (t0 0.52 ± 0.09 µg/µl; t7 0.55 ± 0.05 µg/µl) compared to wildtype mice (t0 0.12 ± 0.03 µg/µl; t7 0.14 ± 0.03 µg/µl), indicating a four- to fivefold increased glycogen concentration in Pompe mice (Fig. S2; t0 p = 0.005, t1 p < 0.001, t7 p < 0.001).

### Pompe mice showed smaller fibre diameter

**Figure 3:**
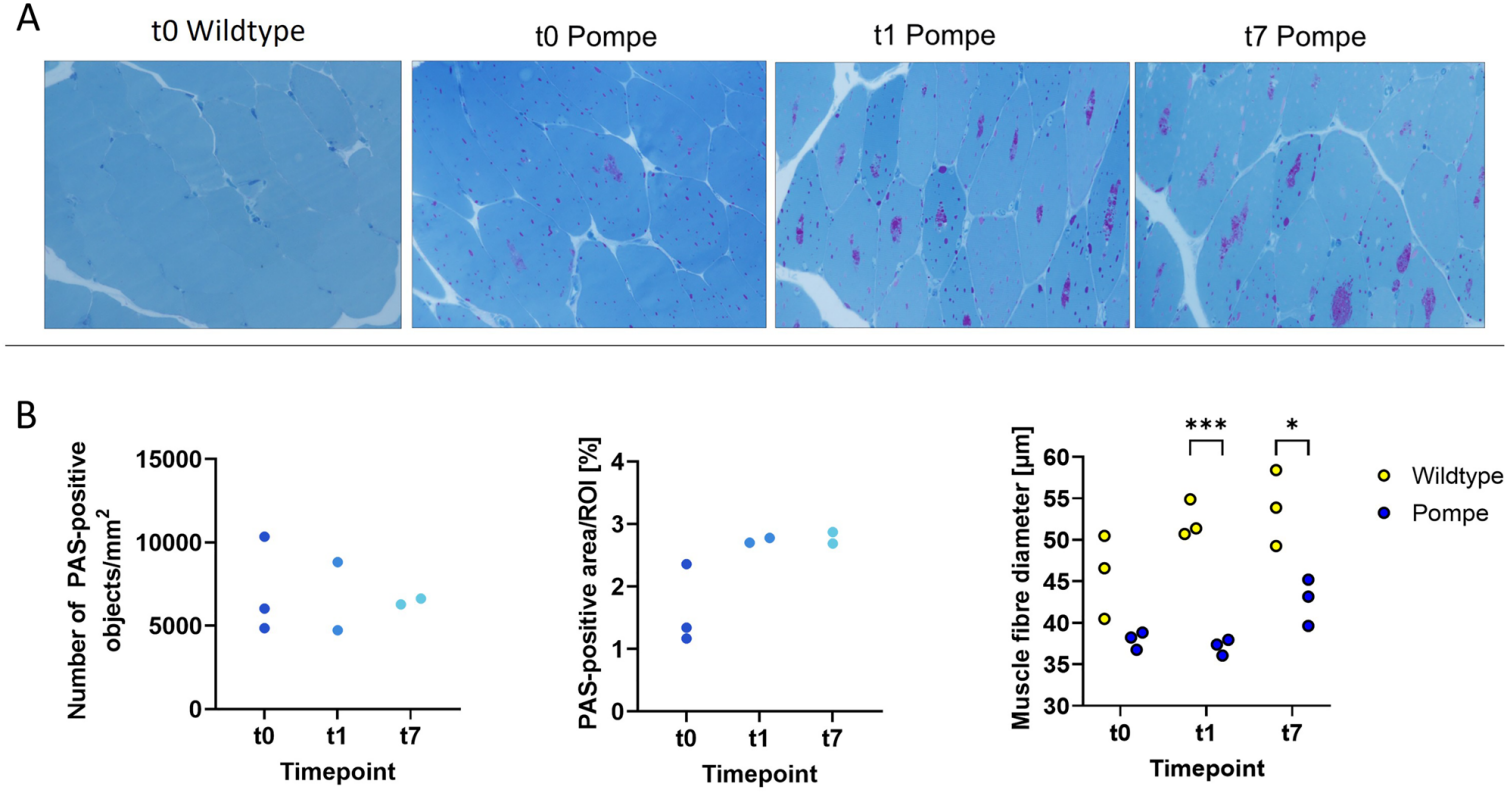
(**A**) PAS-staining for wildtype t0, Pompe t0, t1 and t7. (**B**) The number of PAS-positive objects and areas did not change over t0, t1 and t7, while the fibre diameter of the Pompe quadriceps muscle was significantly smaller at t1 and t7 compared to wildtype (t1 p < 0.001, t7 p = 0.02). Multiple ROIs per mouse were averaged.

Results of the PAS-staining are presented in Fig. 3A. Morphometric analysis of PAS-positive objects and area showed no significant change over time (Fig. 3B; objects/mm^2^: F(2) = 0.03, p = 0.96; area/ROI: F(2) = 5.23, p = 0.07), while the area displayed an insignificant increase from t1 onwards. Pompe and wildtype mice both showed an increase in fibre size over time(not significant using two-way ANOVA: group and timepoint F(1,2) = 1.95, p = 0.18). Focusing on single timepoint differences, Pompe mice showed a significantly smaller quadriceps muscle fibre diameter compared to wildtype mice at t1 and t7 (Fig. 3B; F(1,2) = 1.95, p = 0.18; t0 p = 0.06, t1 p < 0.001, t7 p = 0.02).

### High and continuous LAMP1 abundance is evident, while autophagic markers showed a significant increase over time

**Figure 4:**
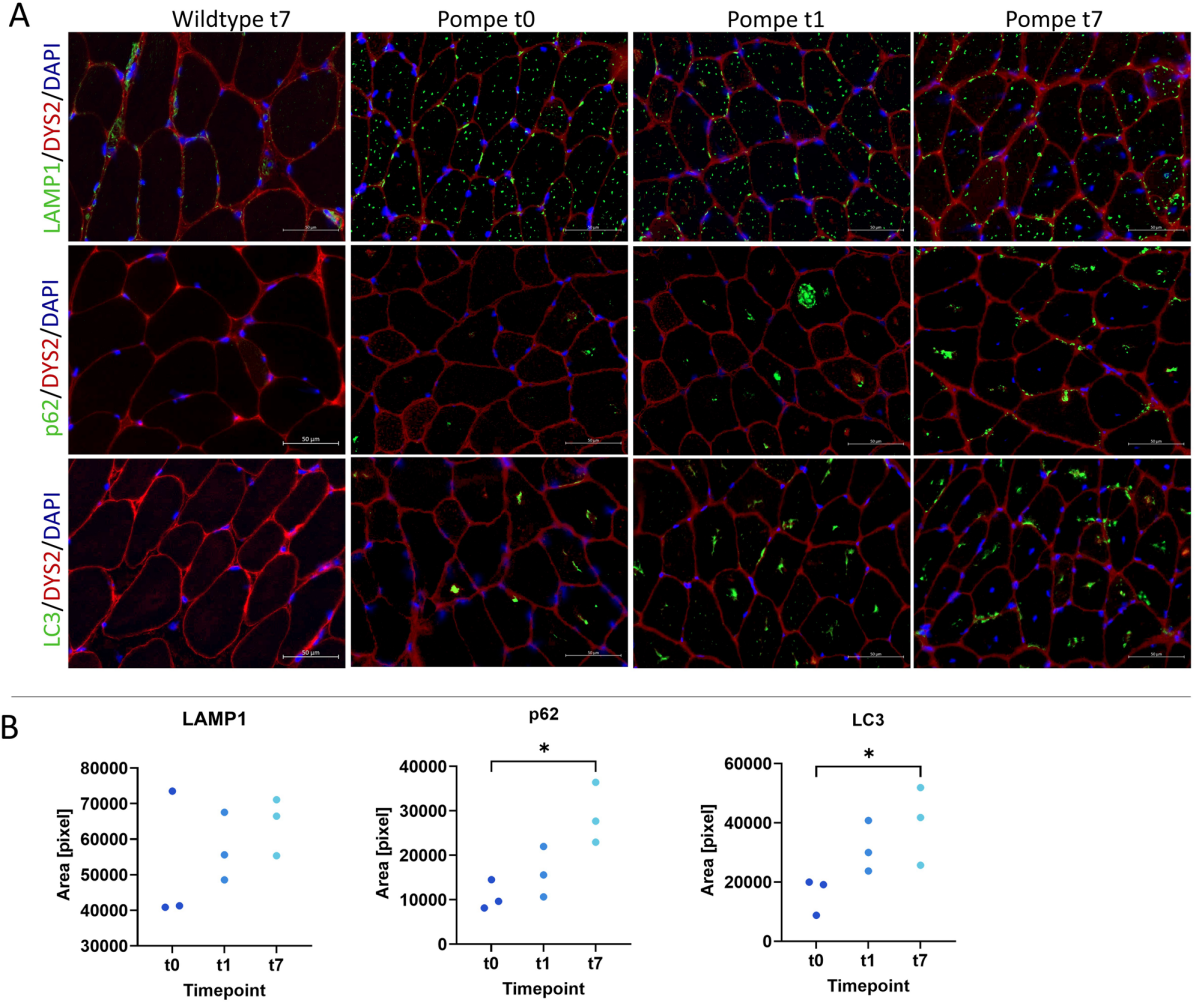
(**A**) Immunofluorescence studies with antibodies against LAMP1, p62 and LC3 with DYS2 and DAPI to display the structure of the cell. (**B**) The area of positive staining for lysosomal marker LAMP1 was not changing from t0 to t1 nor t7. Autophagic markers p62 and LC3 increased significantly when comparing t0 and t7 in Pompe mice. Multiple ROIs per mouse were averaged. Wildtype mice did not show detectable fluorescence intensities for quantification.

The lysosomal membrane protein LAMP1 showed high abundance for all observed timepoints when calculating the area of positive pixels (Fig. 4; for all p > 0.30). However, the autophagic markers p62 and LC3 increased significantly (p62 t0 vs. t7 p = 0.01, LC3 t0 vs. t7 p = 0.04).

### Decrease of diffusion parameters RD and MD correlated with autophagic markers and fibre size in Pompe mice

**Figure 5:**
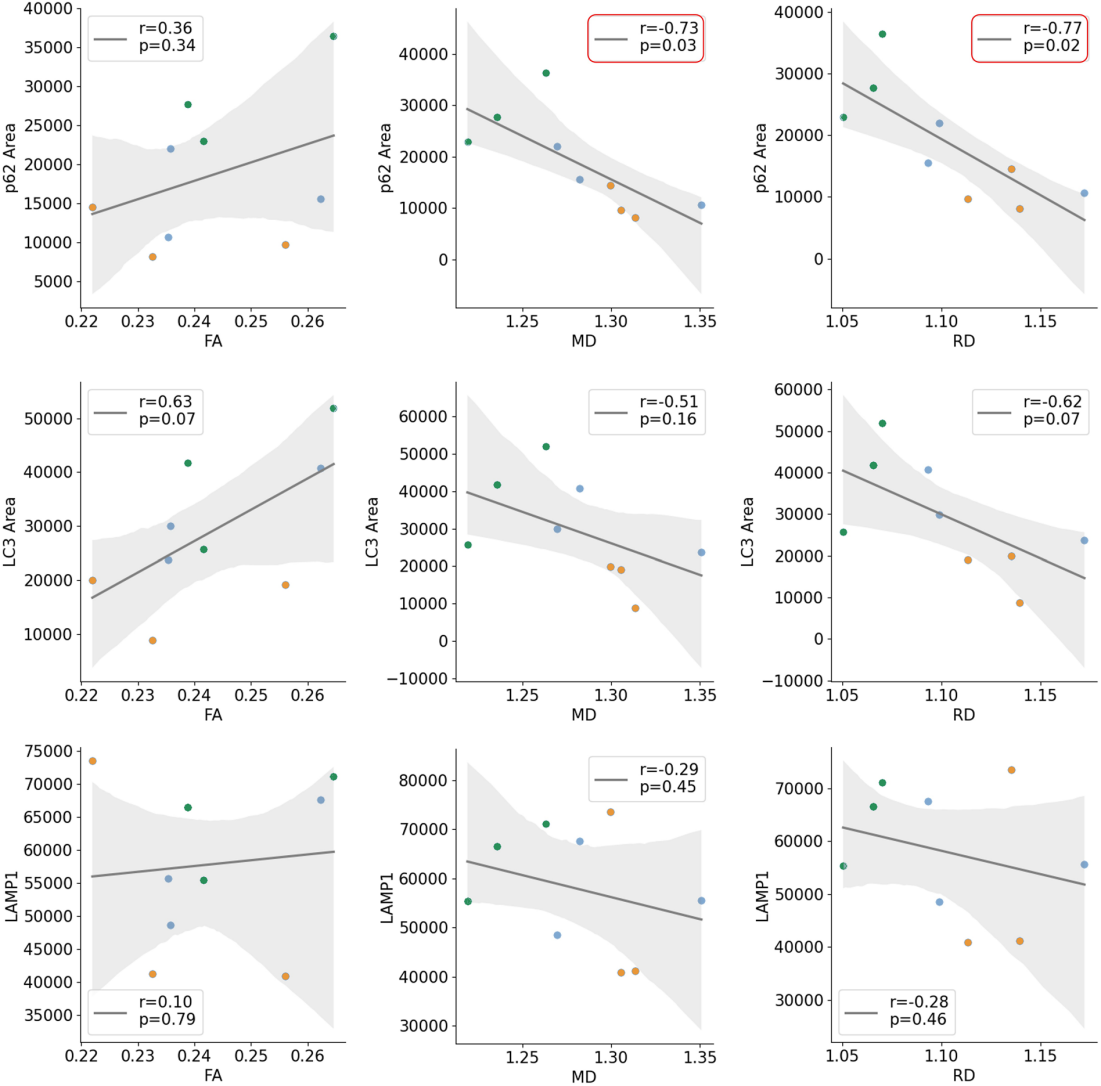
Correlation of diffusion and skeletal muscle pathology in Pompe mice showed significant negative correlations between mean diffusivity (MD) and p62, radial diffusivity (RD) and p62. Orange indicates t0, blue t1 and green t7.

Diffusion parameters FA, MD and RD in quadriceps for the Pompe mice were correlated with histopathological markers at t0, t1 and t7. T-tests showed significant differences in all three parameters in t1 and t7 but not in t0 (Table S2). Therefore, to specifically analyze the development between t0 and t1 shown in diffusion, a correlation analysis between semi-quantitative results of immunofluorescence staining and PAS staining was carried out (Fig. 5).

RD as well as MD both showed significant negative correlations with p62 (r(7) = − 0.77, p = 0.02 and r(7) = − 0.73, p = 0.03). FA did not correlate with p62 (r(7) = 0.36, p = 0.34).

There was no significant correlation of LC3 and FA (r(7) = 0.63, p = 0.07), as well as LC3 with RD and MD (r(7) =  − 0.62, p = 0.07 and r(7) =  − 0.51, p = 0.16).

LAMP1 did not show change from t0 to t7 and there were no significant correlations with any of the diffusion markers (FA/LAMP1 r(7) = 0.10, p = 0.79; RD/LAMP1 and MD/LAMP1 r(7) > − 0.29, p > 0.44).

**Figure 6:**
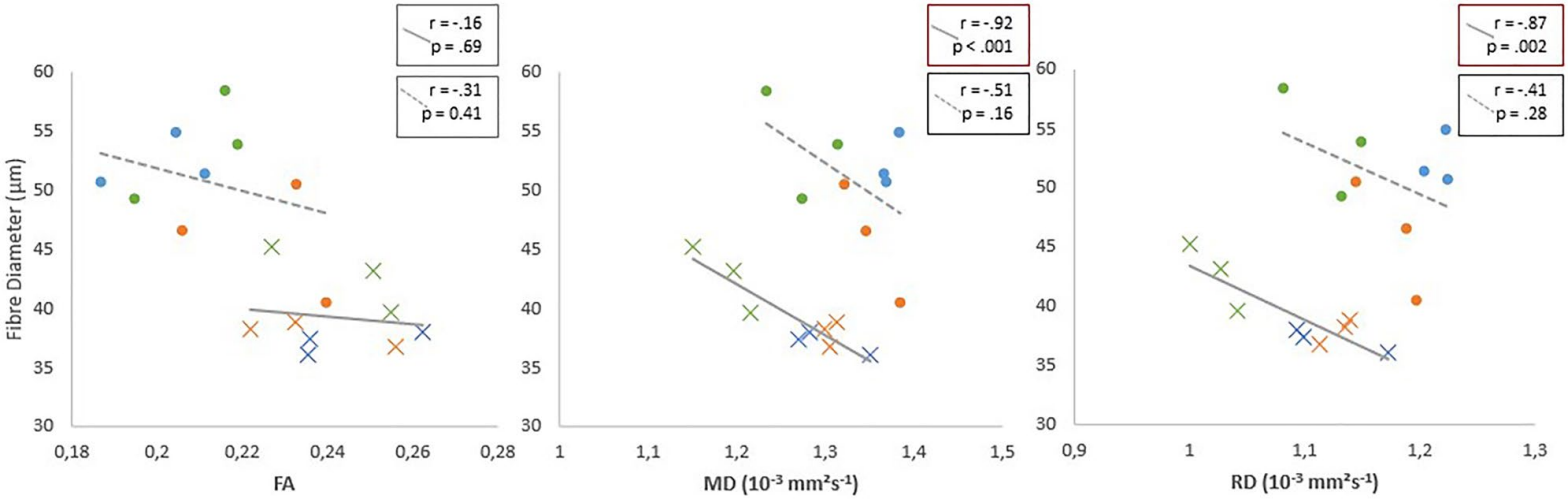
Correlation of fiber diameter and diffusion parameters FA, MD and RD for pompe mice (crosses with solid line) and wildtype mice (dots with dashed line). Orange indicates t0, blue t1 and green t7.

Fibre size negatively correlated with RD and MD (r(7) = − 0.87, p < 0.002; r(7) = − 0.92, p < 0.001) with high significance in pompe mice and non significantly in wildtype mice, while FA did not correlate with fibre size (r(7) = − 0.16, p = 0.69 in pompe) and (r(7) = − 0.31, p = 0.41 in wildtype). See Fig. 6 for details.

## Discussion

In this study, the relationship between qMRI markers and muscle pathology of a Pompe mouse model was investigated over 8 months based on monthly examinations. Significant alterations of water diffusion within the skeletal muscles started at an early age of two months and correlated with hallmarks of skeletal muscle pathology in Pompe disease. Water-T2 and fat fraction were not significantly altered over time and did not differ between groups. Our results indicate that early water diffusion alterations are caused by autophagic buildup and autophagy-related muscle fibre morphology.

In most human studies, alterations in diffusion parameters can be biased by fat^24,25^, since in most patients disease progression is accompanied by fat infiltration. Whereas in wildtype mice, FA decreased and MD, as well as RD, increased, Pompe mice showed a reversed course of increasing FA and decreasing RD and MD in the absence of fat infiltration, which may only develop after symptom onset (after 8 months) and therefore after the timespan of our investigations.

An elevation in FA has been associated with muscle fibre atrophy, as demonstrated by previous studies that correlated histology in various muscular diseases^5,26^ as well as modelled simulations^27^. When the muscle fibre size of Pompe and wildtype mice was compared, we observed that both increase over the observation timeframe, although the diameter of Pompe muscle cells was consistently lower than the wildtype diameter at all investigated timepoints. The increase in fibre size represented in the growing wildtype mice is consistent with the observed decrease of FA. However, an abrupt increase of FA, and decrease of RD and MD, between t0 and t1 in Pompe mice suggests an early change in microstructural skeletal muscle composition that is absent in wildtype mice and not evident in measured fibre diameter of Pompe mice. Although fibre diameter remained stable at t0 and t1 and exhibited a slight increase at t7, the increase in fibre size would suggest an increase of RD and MD, since RD represents the radius of the estimated tensor. In Pompe mice, however, RD and MD decreased although fibre size did not decrease, but slightly increased over time, indicating, that diffusion barriers within the fibres, like autophagic buildup, contributes more to the diffusion signal, than the fibre size itself. Additionally, the utilized short diffusion time (gradient duration / separation  = 3/10 ms) may have been insufficient to fully capture the limits of the fibre boundaries and therefore not be sufficient to represent the fibre size. To validate this, simulations could be of great value. According to literature, altered cell growth might be related to increased activation of autophagy^28^. Interestingly, our study revealed changes in DTI parameters, as early as at the age of two months although the age of onset of symptoms in the Pompe mouse is seven months of age^29^.

While previous studies in LOPD patients revealed diffusion changes in muscles that are not affected by fatty degeneration, the histopathological correlates of these changes remain unsolved^5^. The current hypothesis for the interpretation of qMRI changes in LOPD is that pathological lysosomal glycogen accumulations, caused by GAA deficiency, are responsible for reduced diffusion^5^. However, the present study shows that significant changes in diffusion parameters start with increased autophagic buildup, and not with lysosomal or glycogen abundances. Muscle histology examinations at t0 revealed elevated glycogen content and LAMP1 upregulation had already begun at this timepoint of development, indicating lysosomal and glycogen buildup at an early stage in accordance with previously reported findings^15,30^. Interestingly, when Pompe and wildtype mice were compared, diffusion parameters at t0 remain unchanged, although they clearly diverge at t1, at which time a non-significant elevated LC3 and p62 indicate an early increase in autophagy^10,31,32^. In future studies, it would be interesting to monitor the development of diffusion parameters with therapeutic interventions like ERT, with potential modulators of autophagy or gene therapy in mice.

The variance of qMRI parameters at single timepoints that was observed for individual mice did not influence the statistical significance of each parameter, except for MD in the quadriceps muscle. To unravel the underlying origins of the diffusion changes, the correlation was carried out with quadriceps muscles derived from the Pompe mice at t0, t1 and t7 where results were similar in all muscles. RD and MD correlated significantly with p62 abundance and underline a reduction of radial and overall diffusion due to autophagic buildup.

In contrast to human LOPD studies, water-T2 relaxation time and fat fraction remained unchanged in the Pompe mouse, despite the abovementioned morphological changes^2^. An elevated water-T2 is consistently observed in inflammatory and active muscle degeneration in different myopathies, occurs as the result of tissue oedema and is regarded as an important early marker for active muscle degeneration^33–36^. This could be a crucial factor in the differentiation of disease pathology between mouse models and human LOPD. The disease stages of Pompe mice reflect a large part of the pathophysiology of Pompe disease at clinical and histopathological levels. However, within the timeframe of this study, neither fatty infiltration nor fibrotic muscle changes were observed. Interestingly, in a muscular glycogen storage disease that is associated with a defect in myophosphorylase (Morbus McArdle), and in which intracellular glycogen verifiable accumulates, no autophagic buildup is observed and diffusion changes were also not detected^36^. Taken together, these findings support the results of this study, showing that autophagic buildup rather than glycogen accumulations are revealed by qMRI.

A limitation of our study is the relatively small sample size used for the histological-based analysis of skeletal muscle pathology, since we focused on early changes in t0 to t1 and long-term comparison t7. Nonetheless, significant correlations were found that fit the MRI data. The phenotyping data may have a bias, however, since mice from both genotypes were housed together^37,38^. The IntelliCage setup we used to detect phenotypical changes also might not be sensitive enough to describe the onset of the clinical phenotype of the Pompe mouse model.

In conclusion, this study shows the correlation of changes in diffusion parameters with histopathological alterations in the hind limb muscles of a mouse model for Pompe disease. We infer that changes in diffusion parameters are mainly correlated to autophagic buildup and muscle fibre diameter rather than lysosomal and glycogen accumulations. This finding is important for different aspects regarding Pompe disease: first, it could contribute to the understanding of the underlying pathophysiology of early diffusion changes and clarifies that glycogen accumulation does not correlate with muscle diffusion^36^. Secondly, our findings clearly support the concept that autophagic buildup is an important pathomechanism even in the early stages of disease progression and comprises a major component of disease modifications in Pompe disease.

### Supplementary Information

Supplementary Information.

## Data Availability

Data and further information are available upon request made to lara.schlaffke@rub.de.
