# Supplementary material for: Muscle diffusion MRI reveals autophagic buildup in a mouse model for Pompe disease
Source: Sci Rep. 2023 Dec 20;13:22822. doi: 10.1038/s41598-023-49971-9 (PMC10739793; doi:10.1038/s41598-023-49971-9)
Supplement: Supplementary file 1 — Supplementary Information. [file 41598_2023_49971_MOESM1_ESM.docx]

Supplements

Table 1S: Primary and secondary antibodies used in immunofluorescence studies

| Primary Antibody | Dilution in blocking serum | Manufactory |
| --- | --- | --- |
| Dys2 | 1:50 | NCL-DYS2 mouse [RRID: AB_442081], Novocastra Laboratories, Newcastle on Tyne, UK |
| LC3 | 1:200 | LC3B rabbit [RRID: AB_2827794], ab192890, Abcam, Cambridge, UK |
| p62 | 1:1000 | p62 rabbit [RRID: AB_2810880], ab109012, Abcam, Cambridge, UK |
| LAMP1 | 1:200 | LAMP1 rabbit [RRID: AB_477157], L1418, Sigma, Saint Quentin-Fallavier, France |
| Secondary Antibody | Dilution in blocking serum | Manufactory |
| Alexa green 488 anti-rabbit | 1:1500 | Goat [RRID: AB_2313584], Jackson ImmunoResearch Laboratories, Pennsylvania, USA |
| Alexa red 594 anti-mouse | 1:1000 | Goat [RRID: AB_2338881], Jackson ImmunoResearch Laboratories, Pennsylvania, USA |

Table S2: Mean ± SD values for qMRI results in each muscle with fat fraction (FatFr), water-T2 mapping (T2), fractional anisotropy (FA), mean diffusivity (MD), radial diffusivity (RD) and λ1 for each genotype (GT).

| tp | Muscle | GT | FatFr | T2 | FA | MD | RD | λ1 | GT | FatFr | T2 | FA | MD | RD | λ1 |
| --- | --- | --- | --- | --- | --- | --- | --- | --- | --- | --- | --- | --- | --- | --- | --- |
| t0 | Gastro  Soleus | Pompe | 3.34 ± 0.94 | 21.82 ± 1.19 | 0.23 ± 0.01 | 1.34 ± 0.04 | 1.17 ± 0.04 | 1.69 ± 0.05 | Wildtype | 3.33 ± 0.98 | 20.58 ± 0.48 | 0.22 ± 0.02 | 1.35 ± 0.02 | 1.18 ± 0.03 | 1.69 ± 0.03 |
|  | Tibialis anterior |  | 3.55 ± 1.21 | 20.67 ± 2.20 | 0.26 ± 0.01 | 1.24 ± 0.04 | 1.06 ± 0.03 | 1.62 ± 0.05 |  | 3.38 ± 0.71 | 19.09 ± 1.06 | 0.25 ± 0.01 | 1.25 ± 0.02 | 1.08 ± 0.02 | 1.61 ± 0.03 |
|  | Hamstrings |  | 3.47 ± 0.99 | 21.52 ± 3.49 | 0.22 ± 0.03 | 1.33 ± 0.03 | 1.16 ± 0.04 | 1.68 ± 0.06 |  | 3.02 ± 0.71 | 22.13 ± 1.83 | 0.20 ± 0.03 | 1.35 ± 0.03 | 1.20 ± 0.05 | 1.66 ± 0.03 |
|  | Quadriceps |  | 4.71 ± 1.89 | 21.60 ± 3.17 | 0.24 ± 0.01 | 1.31 ± 0.03 | 1.13 ± 0.04 | 1.67 ± 0.04 |  | 3.56 ± 0.42 | 21.99 ± 1.90 | 0.23 ± 0.01 | 1.35 ± 0.03 | 1.17 ± 0.02 | 1.70 ± 0.04 |
| t1 | Gastro  Soleus | Pompe | 3.55 ± 1.09 | 22.21 ± 1.73 | 0.24 ± 0.02 | 1.31 ± 0.04 | 1.13 ± 0.05 | 1.65 ± 0.04 | Wildtype | 3.64 ± 1.34 | 20.75 ± 2.07 | 0.22 ± 0.01 | 1.40 ± 0.02 | 1.23 ± 0.02 | 1.74 ± 0.03 |
|  | Tibialis anterior |  | 3.14 ± 1.41 | 20.92 ± 1.51 | 0.26 ± 0.01 | 1.23 ± 0.03 | 1.05 ± 0.03 | 1.61 ± 0.03 |  | 3.08 ± 1.19 | 19.39 ± 1.55 | 0.24 ± 0.01 | 1.32 ± 0.02 | 1.14 ± 0.01 | 1.68 ± 0.03 |
|  | Hamstrings |  | 2.95 ± 0.58 | 22.88 ± 1.93 | 0.22 ± 0.02 | 1.27 ± 0.05 | 1.11 ± 0.04 | 1.58 ± 0.08 |  | 2.73 ± 0.57 | 22.67 ± 1.77 | 0.20 ± 0.02 | 1.39 ± 0.02 | 1.24 ± 0.03 | 1.70 ± 0.04 |
|  | Quadriceps |  | 4.04 ± 0.97 | 22.87 ± 2.21 | 0.25 ± 0.01 | 1.27 ± 0.05 | 1.09 ± 0.05 | 1.63 ± 0.06 |  | 3.25 ± 0.72 | 22.89 ± 1.48 | 0.21 ± 0.02 | 1.38 ± 0.01 | 1.21 ± 0.02 | 1.71 ± 0.03 |
| t2 | Gastro  Soleus | Pompe | 4.08 ± 1.36 | 19.92 ± 1.60 | 0.25 ± 0.01 | 1.27 ± 0.02 | 1.09 ± 0.03 | 1.61 ± 0.03 | Wildtype | 3.24 ± 1.00 | 20.39 ± 0.68 | 0.22 ± 0.01 | 1.36 ± 0.02 | 1.19 ± 0.02 | 1.69 ± 0.03 |
|  | Tibialis anterior |  | 4.20 ± 2.24 | 18.09 ± 1.21 | 0.26 ± 0.01 | 1.19 ± 0.01 | 1.01 ± 0.01 | 1.55 ± 0.03 |  | 2.95 ± 1.15 | 18.88 ± 1.00 | 0.23 ± 0.01 | 1.29 ± 0.02 | 1.12 ± 0.01 | 1.64 ± 0.03 |
|  | Hamstrings |  | 3.25 ± 0.83 | 21.74 ± 1.75 | 0.24 ± 0.02 | 1.24 ± 0.04 | 1.07 ± 0.05 | 1.59 ± 0.05 |  | 2.74 ± 0.63 | 20.08 ± 2.96 | 0.20 ± 0.02 | 1.38 ± 0.02 | 1.22 ± 0.03 | 1.70 ± 0.04 |
|  | Quadriceps |  | 3.33 ± 0.20 | 22.35 ± 1.73 | 0.25 ± 0.02 | 1.25 ± 0.03 | 1.07 ± 0.03 | 1.60 ± 0.04 |  | 3.20 ± 0.27 | 20.41 ± 2.41 | 0.21 ± 0.01 | 1.36 ± 0.03 | 1.20 ± 0.02 | 1.68 ± 0.05 |
| t3 | Gastro  Soleus | Pompe | 3.06 ± 1.13 | 20.77 ± 1.89 | 0.25 ± 0.01 | 1.26 ± 0.04 | 1.08 ± 0.04 | 1.61 ± 0.05 | Wildtype | 3.81 ± 1.40 | 20.01 ± 1.13 | 0.22 ± 0.01 | 1.34 ± 0.03 | 1.18 ± 0.03 | 1.67 ± 0.04 |
|  | Tibialis anterior |  | 2.53 ± 0.91 | 19.52 ± 1.37 | 0.27 ± 0.01 | 1.19 ± 0.04 | 1.00 ± 0.03 | 1.56 ± 0.07 |  | 3.41 ± 1.62 | 18.80 ± 1.16 | 0.23 ± 0.01 | 1.28 ± 0.02 | 1.11 ± 0.02 | 1.62 ± 0.03 |
|  | Hamstrings |  | 2.76 ± 0.84 | 22.49 ± 2.08 | 0.24 ± 0.03 | 1.25 ± 0.05 | 1.08 ± 0.05 | 1.59 ± 0.07 |  | 3.14 ± 0.72 | 21.68 ± 1.64 | 0.19 ± 0.01 | 1.37 ± 0.02 | 1.23 ± 0.03 | 1.66 ± 0.02 |
|  | Quadriceps |  | 3.66 ± 0.65 | 23.02 ± 1.39 | 0.26 ± 0.01 | 1.25 ± 0.05 | 1.06 ± 0.04 | 1.61 ± 0.07 |  | 3.83 ± 0.44 | 21.60 ± 2.06 | 0.22 ± 0.01 | 1.36 ± 0.01 | 1.19 ± 0.01 | 1.69 ± 0.02 |
| t4 | Gastro  Soleus | Pompe | 4.00 ± 0.89 | 20.51 ± 1.96 | 0.24 ± 0.02 | 1.24 ± 0.06 | 1.07 ± 0.06 | 1.57 ± 0.06 | Wildtype | 3.29 ± 1.22 | 19.47 ± 0.75 | 0.21 ± 0.01 | 1.35 ± 0.04 | 1.19 ± 0.03 | 1.67 ± 0.05 |
|  | Tibialis anterior |  | 3.68 ± 1.24 | 19.13 ± 1.91 | 0.26 ± 0.01 | 1.17 ± 0.03 | 0.99 ± 0.03 | 1.52 ± 0.04 |  | 2.78 ± 1.03 | 18.09 ± 1.42 | 0.23 ± 0.01 | 1.27 ± 0.03 | 1.10 ± 0.02 | 1.60 ± 0.03 |
|  | Hamstrings |  | 3.27 ± 0.66 | 22.58 ± 1.47 | 0.24 ± 0.02 | 1.26 ± 0.06 | 1.08 ± 0.05 | 1.61 ± 0.10 |  | 2.78 ± 0.78 | 22.53 ± 1.73 | 0.19 ± 0.02 | 1.36 ± 0.03 | 1.21 ± 0.04 | 1.66 ± 0.04 |
|  | Quadriceps |  | 3.90 ± 0.18 | 23.47 ± 1.70 | 0.25 ± 0.03 | 1.26 ± 0.05 | 1.08 ± 0.06 | 1.62 ± 0.07 |  | 3.80 ± 0.81 | 22.17 ± 0.90 | 0.20 ± 0.01 | 1.36 ± 0.03 | 1.20 ± 0.03 | 1.67 ± 0.04 |
| t5 | Gastro  Soleus | Pompe | 3.36 ± 1.17 | 20.75 ± 2.21 | 0.24 ± 0.02 | 1.26 ± 0.05 | 1.10 ± 0.06 | 1.59 ± 0.04 | Wildtype | 3.15 ± 1.04 | 19.52 ± 1.48 | 0.21 ± 0.01 | 1.36 ± 0.02 | 1.20 ± 0.02 | 1.68 ± 0.03 |
|  | Tibialis anterior |  | 3.34 ± 1.06 | 18.79 ± 2.97 | 0.26 ± 0.02 | 1.19 ± 0.06 | 1.01 ± 0.07 | 1.54 ± 0.05 |  | 2.93 ± 0.97 | 17.50 ± 0.76 | 0.23 ± 0.00 | 1.26 ± 0.02 | 1.10 ± 0.02 | 1.59 ± 0.03 |
|  | Hamstrings |  | 2.94 ± 0.69 | 22.59 ± 2.41 | 0.25 ± 0.01 | 1.24 ± 0.05 | 1.06 ± 0.04 | 1.60 ± 0.07 |  | 2.85 ± 0.68 | 22.80 ± 1.75 | 0.20 ± 0.02 | 1.35 ± 0.02 | 1.20 ± 0.02 | 1.65 ± 0.05 |
|  | Quadriceps |  | 3.62 ± 0.98 | 22.75 ± 1.96 | 0.24 ± 0.02 | 1.23 ± 0.03 | 1.06 ± 0.04 | 1.57 ± 0.04 |  | 3.59 ± 0.15 | 22.65 ± 1.25 | 0.20 ± 0.01 | 1.35 ± 0.03 | 1.20 ± 0.03 | 1.66 ± 0.03 |
| t6 | Gastro  Soleus | Pompe | 3.35 ± 0.98 | 19.47 ± 1.74 | 0.25 ± 0.01 | 1.26 ± 0.03 | 1.09 ± 0.03 | 1.62 ± 0.04 | Wildtype | 4.17 ± 0.70 | 19.20 ± 1.22 | 0.21 ± 0.01 | 1.37 ± 0.03 | 1.22 ± 0.03 | 1.69 ± 0.04 |
|  | Tibialis anterior |  | 3.45 ± 0.78 | 18.64 ± 1.58 | 0.27 ± 0.01 | 1.16 ± 0.01 | 0.98 ± 0.01 | 1.53 ± 0.01 |  | 3.91 ± 0.95 | 17.97 ± 1.43 | 0.23 ± 0.01 | 1.30 ± 0.02 | 1.13 ± 0.01 | 1.64 ± 0.04 |
|  | Hamstrings |  | 3.13 ± 0.72 | 20.92 ± 2.01 | 0.26 ± 0.01 | 1.21 ± 0.02 | 1.03 ± 0.02 | 1.58 ± 0.03 |  | 3.53 ± 0.57 | 21.61 ± 1.93 | 0.20 ± 0.02 | 1.38 ± 0.03 | 1.22 ± 0.04 | 1.69 ± 0.04 |
|  | Quadriceps |  | 4.01 ± 0.49 | 21.51 ± 1.77 | 0.26 ± 0.02 | 1.26 ± 0.02 | 1.07 ± 0.02 | 1.63 ± 0.05 |  | 4.36 ± 0.75 | 20.50 ± 4.27 | 0.20 ± 0.02 | 1.40 ± 0.04 | 1.24 ± 0.03 | 1.71 ± 0.07 |
| t7 | Gastro  Soleus | Pompe | 3.86 ± 1.01 | 19.80 ± 1.21 | 0.24 ± 0.01 | 1.23 ± 0.03 | 1.07 ± 0.03 | 1.55 ± 0.05 | Wildtype | 3.39 ± 1.05 | 18.51 ± 0.73 | 0.20 ± 0.01 | 1.33 ± 0.04 | 1.18 ± 0.04 | 1.63 ± 0.05 |
|  | Tibialis anterior |  | 3.73 ± 1.51 | 17.81 ± 0.65 | 0.27 ± 0.01 | 1.14 ± 0.03 | 0.96 ± 0.02 | 1.50 ± 0.04 |  | 3.02 ± 0.66 | 16.85 ± 1.35 | 0.23 ± 0.01 | 1.24 ± 0.04 | 1.08 ± 0.04 | 1.57 ± 0.05 |
|  | Hamstrings |  | 2.97 ± 0.87 | 20.95 ± 2.16 | 0.28 ± 0.02 | 1.18 ± 0.05 | 0.99 ± 0.05 | 1.56 ± 0.06 |  | 2.65 ± 0.68 | 21.64 ± 1.22 | 0.20 ± 0.02 | 1.30 ± 0.06 | 1.16 ± 0.06 | 1.60 ± 0.05 |
|  | Quadriceps |  | 4.01 ± 1.26 | 21.47 ± 2.20 | 0.25 ± 0.01 | 1.21 ± 0.04 | 1.04 ± 0.03 | 1.56 ± 0.06 |  | 3.56 ± 0.45 | 21.64 ± 1.22 | 0.21 ± 0.01 | 1.32 ± 0.06 | 1.16 ± 0.06 | 1.63 ± 0.08 |


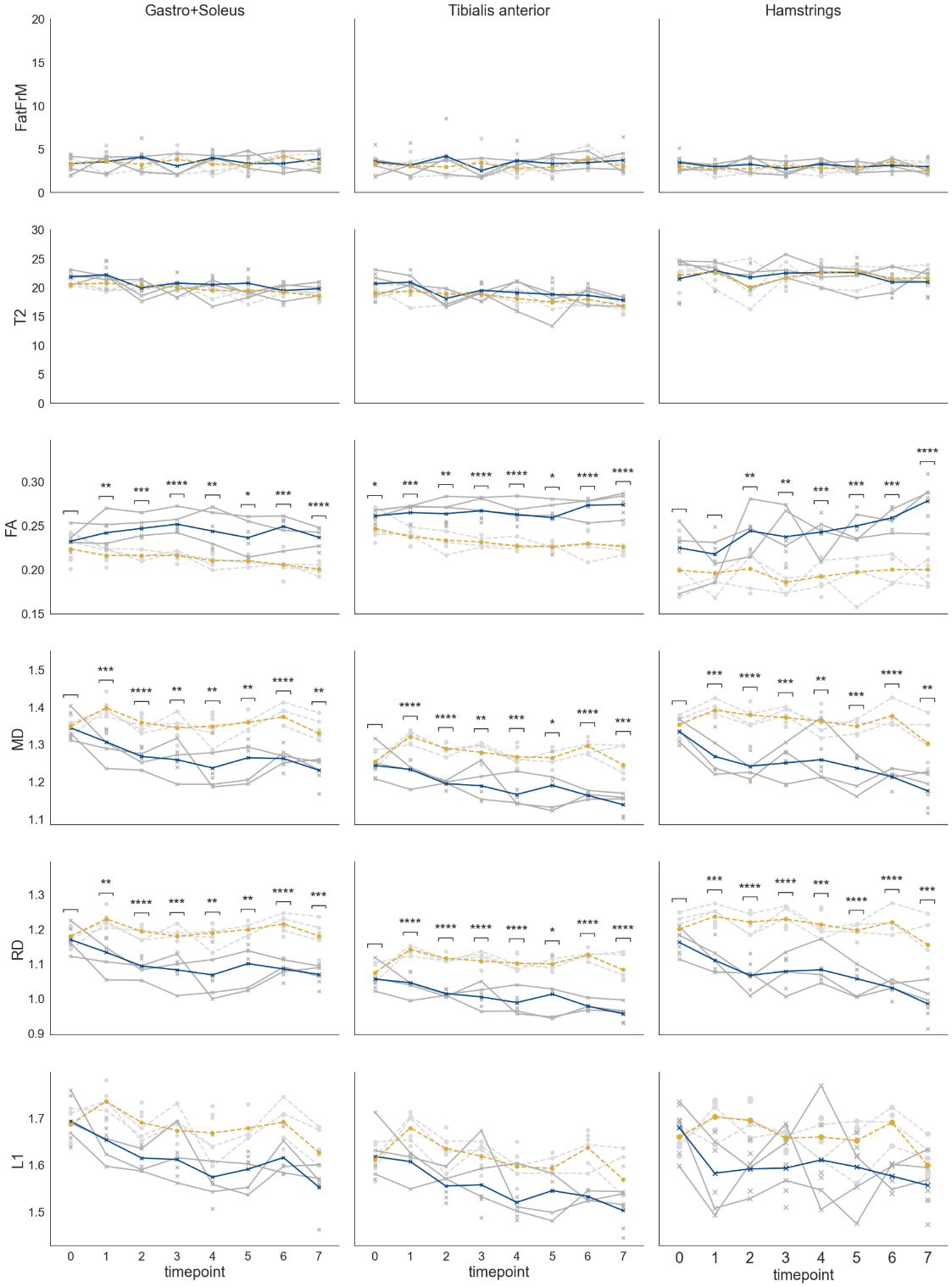


Figure S1: Upper and lower leg muscles DTI, T2-mapping and Dixon-based water fat imaging results. While fat fraction (FatFr) and T2 were not significant different comparing Pompe and wildtype mice, the diffusion parameters fractional anisotropy (FA), mean diffusivity (MD) and radial diffusivity (RD) showed significant changes. FA increased over the observed timespan, while MD and RD decreased. λ_1_ showed a decreasing trend but was not significant.


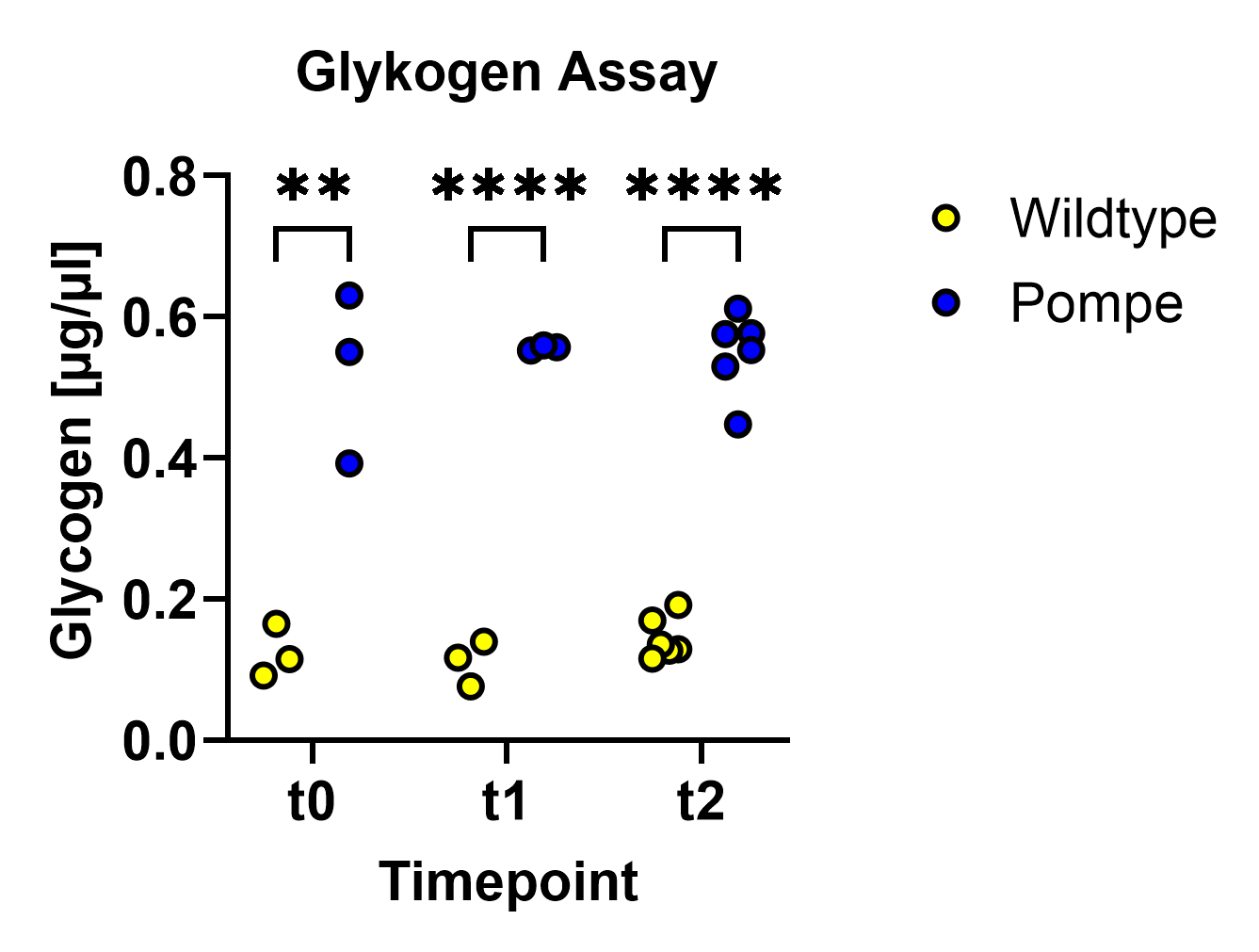


Figure S2: Glycogen content measured with glycogen assay. Pompe mice show a high concentration of glycogen over the observed timecourse
